# Development of a novel in‐house blood biomarker panel for early diagnosis of Alzheimer’s disease

**DOI:** 10.1002/alz70861_108325

**Published:** 2025-12-23

**Authors:** Prita Riana Asih, Juan Lantero Rodriguez, Steve Pedrini, EMS Bandara, Kristie Stefanoska, Henrik Zetterberg, Arne Ittner, Kaj Blennow, Ralph N Martins

**Affiliations:** ^1^ Macquarie University, Sydney, NSW Australia; ^2^ Alzheimer's Research Australia (ARA), Nedlands, Western Australia Australia; ^3^ Lions Alzheimer's Foundation, Nedlands, Western Australia Australia; ^4^ Clinical Neurochemistry Laboratory, Sahlgrenska University Hospital, Mölndal Sweden; ^5^ Institute of Neuroscience & Physiology, Department of Psychiatry & Neurochemistry, The Sahlgrenska Academy, University of Gothenburg, Mölndal Sweden; ^6^ Australian Alzheimer’s Research Foundation, Perth, Western Australia Australia; ^7^ Edith Cowan University, Perth, Western Australia Australia; ^8^ Flinders University, Bedford Park, SA Australia; ^9^ Hong Kong Center for Neurodegenerative Diseases, Hong Kong, Science Park China; ^10^ Theme Aging, Karolinska University Hospital, Stockohlm Sweden; ^11^ Clinical Neurochemistry Laboratory, Sahlgrenska University Hospital, Mölndal, Västra Götalands län Sweden; ^12^ Department of Neurodegenerative Disease, National Hospital for Neurology and Neurosurgery, UCL Institute of Neurology, London UK; ^13^ University of Wisconsin‐Madison, Madison, WI USA; ^14^ Universitat Pompeu Fabra, Barcelona Spain; ^15^ Department of Psychiatry and Neurochemistry, Institute of Neuroscience and Physiology, The Sahlgrenska Academy, University of Gothenburg, Mölndal Sweden; ^16^ Karolinska Institutet, Stockholm Sweden; ^17^ Paris Brain Institute, ICM, Pitié‐Salpêtrière Hospital, Sorbonne Université, Paris France; ^18^ Neurodegenerative Disorder Research Center, Division of Life Sciences and Medicine, Institute on Aging and Brain Disorders, University of Science and Technology of China and First Affiliated Hospital of USTC, 合肥, 安徽 China; ^19^ Stark Neurosciences Research Institute, Indiana University School of Medicine, Indianapolis, IN USA; ^20^ Alzheimer's Research Australia, Perth, Western Australia Australia; ^21^ Lions Alzheimer's Foundation, Perth, Western Australia Australia; ^22^ Centre for Ageing, Cognition and Wellbeing, Macquarie University, North Ryde, NSW Australia

## Abstract

**Background:**

Currently a definitive diagnosis for Alzheimer’s disease (AD) is not readily available to the wider community as the gold standard markers of AD are either invasive and/or expensive. Therefore, there is an urgent need for a relatively low‐cost blood test that can be readily used in clinical settings. This study aims to address this gap by developing a reliable and cost‐effective in‐house blood‐based biomarkers panel for routine use in clinical labs for early detection of AD. Our team have shown that specific proteins in the blood, namely phosphorylated tau and Glial Fibrillary Acidic Protein (GFAP) as biomarker for early AD diagnosis. However, only a combination of selected blood biomarkers can reliably predict who is at risk of AD. To achieve a combination diagnostic assay based on robust blood biomarkers, this study will first develop singleplex diagnostic Single Molecule Array (Simoa) blood biomarkers assays for phosphorylated Tau: *p* ‐Tau217 and pTau205, as well as the N‐ and C‐terminus of GFAP. A novel panel of the best 3 biomarkers will then be selected to establish a blood test for preclinical diagnosis of AD.

**Method:**

Monoclonal antibodies against *p* ‐Tau217, *p* ‐Tau205, N‐ and C‐terminus of GFAP were generated in collaboration with GenScript, Singapore. These antibodies have undergone initial characterisation with controlled primary assessment of specificity and affinity (i.e immunostaining, immunoprecipitation‐Western Blot, and immunoprecipitation‐mass spectrometry) using various tissues such as human cells expressing phospho‐site‐deficient tau as negative controls, human brain, CSF, and plasma from healthy controls and AD. Finally, these antibodies were developed for Simoa assay in collaboration with University of Gothenburg.

**Result:**

We have successfully generated specific high affinity monoclonal antibodies against *p* ‐Tau217, *p* ‐Tau205, and N‐terminus GFAP and have confirmed that these antibodies have the necessary specificity and sensitivity for the Simoa platform.

**Conclusion:**

The development of Simoa assays will be validated in a highly characterised control and Alzheimer cohort The Australian Imaging, Biomarker, and Lifestyle (AIBL) and a novel panel of the best 3 biomarkers established.

